# HIV infection as a risk factor for shigellosis.

**DOI:** 10.3201/eid0506.990614

**Published:** 1999

**Authors:** J T Baer, D J Vugia, A L Reingold, T Aragon, F J Angulo, W Z Bradford

**Affiliations:** California Emerging Infections Program, San Francisco, California, USA

## Abstract

We investigated cases of shigellosis in San Francisco and Alameda Counties identified during 1996 by active laboratory surveillance to assess the role of HIV infection as a risk factor for shigellosis. Dramatically elevated rates of shigellosis in HIV-infected persons implicate HIV infection as an important risk factor for shigellosis in San Francisco.


### HIV Infection as a Risk Factor for Shigellosis[1]

**Jefferson T. Baer,* Duc J. Vugia,*†‡ Arthur L. Reingold ,*§ Tomas Aragon,¶ Frederick J. Angulo,# and Williamson Z. Bradford*§****

*California Emerging Infections Program, San Francisco, California, USA; †California Department of Health Services, Berkeley, California, USA; ‡University of California, San Francisco, California, USA; §School of Public Health, University of California, Berkeley, California, USA; ¶San Francisco Department of Public Health, San Francisco, California, USA; #Centers for Disease Control and Prevention, Atlanta, Georgia, USA; and **IntraBiotics Pharmaceuticals, Mountain View, California, USA

We investigated cases of shigellosis in San Francisco and Alameda Counties identified during 1996 by active laboratory surveillance to assess the role of HIV infection as a risk factor for shigellosis. Dramatically elevated rates of shigellosis in HIV-infected persons implicate HIV infection as an important risk factor for shigellosis in San Francisco.

*Shigella* infections are responsible for an estimated 300,000 illnesses and 600 deaths per year in the United States and more than 600,000 deaths per year worldwide (1). *Shigella* species are typically transmitted by direct or indirect fecal-oral contact; as a result, shigellosis has long been associated with outbreaks in day-care centers, nursing homes, and institutionalized populations (2-4). However, several studies have demonstrated an increased frequency of shigellosis cases in young adult men residing in urban settings who have little, if any, exposure to these traditionally recognized risk groups (5-9). These investigations also suggest that *Shigella* infection occurs during the practice of gay sex; however, since most of these studies occurred before the HIV epidemic, the relationship between HIV infection and gay sex and the subsequent risk for shigellosis has yet to be evaluated (5-9).

HIV-infected persons are at increased risk for infection by several common enteric pathogens (10). Previous investigations have demonstrated that HIV-infected patients are at 20 times greater risk for infection with *Salmonella* species and 39 times greater risk for infection with *Campylobacter* species than the general population (11,12). To determine the rate of shigellosis in HIV-infected persons, we investigated all cases of shigellosis in San Francisco, a county with a high prevalence of HIV infection and a high incidence of shigellosis. Alameda, a neighboring county with lower rates of shigellosis and HIV infection, was used as a comparison area for our investigation because of its proximity and differing shigellosis and HIV epidemiology.

During 1996, cases of culture-confirmed shigellosis were identified in San Francisco and Alameda Counties by active surveillance in 28 laboratories for isolates of *Shigella* species cultured from any anatomic site as part of the California Emerging Infections Program. The program comprises one of five sites in the Foodborne Diseases Active Surveillance Network (FoodNet), which is part of the Centers for Disease Control and Prevention's Emerging Infections Program. All available medical records of patients were reviewed by a standardized data collection instrument detailing demographic and medical information. Data concerning sexual activity and orientation and foreign travel were obtained from routine telephone interviews of patients with shigellosis, conducted by the San

Address for correspondence: Williamson Z. Bradford, IntraBiotics Pharmaceuticals, Inc., 1255 Terra Bella Ave., Mountain View, CA 94080, USA; fax: 650-969-0663; e-mail: BBradford@intrabiotics.com.

[1]Presented in part at the International Conference on Emerging Infections, March 1998, Atlanta, Georgia.

Francisco Department of Public Health. This information was not available for Alameda residents because no telephone interviews were conducted in Alameda County.

Patients were considered HIV-infected if their medical record contained a physician's note or a laboratory report documenting HIV infection. In the absence of such documentation, patients were considered HIV-negative. San Francisco patients were classified as gay if they were male and had identified themselves as gay or bisexual during telephone interviews. Recent sexual contact for San Francisco patients was assessed during the telephone interviews and was defined as having had a sexual encounter within 10 days of the onset of shigellosis symptoms. Foreign travel exposure was defined as travel to an area where shigellosis was endemic 7 days before the onset of symptoms.

Postcensus data for San Francisco and Alameda Counties were obtained for 1996 (13). Estimates of the prevalence of HIV infection in San Francisco by groups at risk were obtained from the 1997 HIV Consensus Report on HIV Prevalence and Incidence in San Francisco (14). This report is based on the findings of a consensus panel that systematically reviewed numerous sources of published and unpublished data. FoodNet incidence rates for culture-confirmed cases of shigellosis were based on aggregated data collected by active laboratory-based surveillance during 1996 in four urban areas in Connecticut, Georgia, Minnesota, and Oregon (15). National incidence rates were based on culture-confirmed cases of shigellosis reported through passive laboratory-based surveillance in 1996 (15).

Data were managed and analyzed by Stata 4.0 (Stata Corporation, College Station, TX) and EpiInfo 6.04b (CDC, Atlanta, GA) software. Univariate analyses of proportions were performed by chi-square test. Incidence rates and incidence rate ratios were compared by using the exact method to calculate confidence intervals and statistical significance. Temporal trends of shigellosis were assessed by conducting chi-square for trend analysis on the number of cases of shigellosis diagnosed by month.

During 1996, 228 and 140 culture-confirmed cases of shigellosis were identified in San Francisco and Alameda, respectively. In San Francisco, 142 (62%) of these cases were caused by *S. sonnei*, 73 (32%) by *S. flexneri*, 7 (3%) by *S. boydi*, 2 (1%) by *S. dysenteriae,* and 4 (2%) were not speciated. In Alameda, 93 (66%) of the cases were caused by *S. sonnei*, 28 (20%) by *S. flexneri*, 6 (4%) by *S. boydi*, 2 (2%) by *S. dysenteriae,* and 11 (8%) were not speciated. No difference was observed in the proportion of cases caused by different species in the two counties (p = 0.16).

An analysis of the month of diagnosis for all patients with *S. sonnei* infections demonstrated a distinct trend in both San Francisco (p = .001) and Alameda (p = .03). The number of infections per month was highest in San Francisco between January and May and in Alameda between August and November. No temporal trend was apparent for *S. flexneri* infections in San Francisco (p = .77) or Alameda (p = .36).

San Francisco patients were significantly more likely than Alameda patients to be male, adult, white, and HIV-infected (Table 1). Sixty-six (39%) of 168 shigellosis patients and 30 (54%) of 56 *S. flexneri*-infected patients in San Francisco were HIV-infected; 11 (15%) of 75 HIV-negative patients and 1 (2%) of 56 HIV-infected patients in San Francisco reported recent travel to a shigella-endemic area outside the United States (p = 0.01).

Table 1. Persons with shigellosis, by county of residence, 1996

| Characteristic | San Francisco (n = 228) | Alameda (n = 140) | p value |
|---|---|---|---|
| Male | 157/228 (69%) | 60/140 (43%) | <0.001 |
| Age ≥18 | 181/227 (80%) | 57/140 (41%) | <0.001 |
| Race | | | |
| White | 117/200 (59%) | 15/92 (16%) | <0.001 |
| Black | 39/200 (20%) | 44/92 (48%) | <0.001 |
| Hispanic | 37/200 (19%) | 22/92 (24%) | 0.285 |
| Hospitalized | 22/201 (11%) | 12/140 (9%) | 0.472 |
| HIV infection | 66/168 (39%) | 9/125 (7%) | <0.001 |
| Foreign travel | 25/185 (14%) | NA[a] | --- |
| Gay male | 96/190 (51%) | NA[a] | --- |
| Recent sex | 70/136 (51%) | NA[a] | --- |

[a]NA = not available

The annual incidence rates of shigellosis for various population groups in San Francisco, Alameda, other FoodNet sites, and the United States are shown in Table 2. San Francisco had higher overall rates, particularly among men and persons ages 25 to 64 years, than Alameda, other FoodNet sites, and the United States.

In San Francisco, an analysis of the annual incidence rates of shigellosis per 100,000

Table 2. Annual incidence rates of culture-confirmed shigellosis per 100,000 population for selected groups, 1996

| Group | San Francisco County | Alameda County | FoodNet sites[a] | United States |
|---|---|---|---|---|
| Overall rate | 30.9 | 10.5[b] | 7.3 | 5.3 |
| Male | 43.2 | 9.2[b] | 7.4 | 3.3 |
| Female | 19.1 | 11.8[b] | 7.1 | 3.9 |
| Age groups (yrs) | | | | |
| <5 | 82.5 | 47.9[b] | 36.7 | 16.7 |
| 5-14 | 22.1 | 17.1 | 12.6 | 6.8 |
| 15-24 | 19.9 | 9.3[b] | 5.3 | 2.4 |
| 25-39 | 49.9 | 7.6[b] | 5.6 | 2.7 |
| 40-64 | 25.9 | 3.3[b] | 2.4 | 1.0 |
| 65+ | 2.5 | 4.0 | 1.5 | 0.7 |

[a]Does not include California.
[b]Comparison of rates in San Francisco and Alameda Counties, p value <.05.

population by sexual orientation and HIV status showed rates of 12.4 in heterosexual and HIV-negative persons, 60.1 in gay and HIV-negative persons, 378 in not gay and HIV-infected persons, and 442 in gay and HIV-infected persons. Incidence rate ratios for these groups, relative to the not gay and HIV-negative population, were as follows: gay and HIV-negative 4.9 (95% CI 2.7-8.1); not gay and HIV-infected 30.6 (95% CI 12.8-63.0); and gay and HIV-infected 35.7 (95% CI 25.1-50.4).

Thirty-four (10%) of 341 of patients were hospitalized for shigellosis (median hospital stay 3 days). Furthermore, in San Francisco 13 (22%) of 60 HIV-infected shigellosis patients were hospitalized, while 7 (8%) of 86 HIV-negative persons were hospitalized (p = .02). Twelve (13%) of 93 *S. flexneri* and 19 (9%) of 216 *S. sonnei* patients were hospitalized (p = .27).

These population-based data demonstrate a high overall annual incidence rate of shigellosis in San Francisco compared with neighboring Alameda County, other FoodNet sites, and the United States, and dramatically elevated rates in HIV-infected San Francisco residents. The high proportion of cases in San Francisco in both the gay and the HIV-infected populations suggests that these groups play a major role in the epidemiologic features of endemic shigellosis in San Francisco. Furthermore, the greatly elevated incidence rates of shigellosis in the HIV-infected population suggest that HIV may be a important risk factor for *Shigella* infection. These data also demonstrate that shigellosis is associated with extensive illness and increased health-care expenditures, particularly in the HIV-infected population, as evidenced by the frequency of hospitalization in these persons.

There are several possible explanations for the high rates of shigellosis observed in HIV-infected patients in this study. The compromised host immunity of HIV-infected persons may increase their risk for clinical infection after exposure. A recent study found that 75% of asymptomatic household contacts of symptomatic shigellosis patients had evidence of *Shigella* infection by polymerase chain reaction, which suggests that host immunity may play an important role in determining which exposed persons progress to clinical infection (16). Increased susceptibility to shigellosis among HIV-infected persons could be mediated through different mechanisms, including compromised cell-mediated immunity or achlorhydria. Alternatively, the high rate of shigellosis in HIV-infected patients may be related to factors other than host immunity, such as sexual or behavioral practices, which were not thoroughly investigated in our study.

Our investigation suggests that HIV infection is an important risk factor for shigellosis. This finding has not been previously described on a population level. This investigation also suggests that HIV infection is an important determinant of the epidemiologic features of shigellosis in San Francisco and that public health prevention strategies in areas with a large HIV-infected and gay male population may need to be revisited. A diagnosis of shigellosis in young adult men who are not part of a recognized outbreak and have not recently traveled to a *Shigella*-endemic area may serve as a marker for HIV infection and may indicate a need for counseling and HIV testing.

Due to the methods of data collection, misclassification of HIV infection status and sexual orientation could have occurred in either shigellosis patients or in the San Francisco Department of Public Health population estimates, thereby altering the incidence rates in an unpredictable manner. Moreover, the propensity of HIV-infected patients with shigellosis to seek medical attention and the likelihood of their health-care providers to obtain cultures may differ from that of HIV-negative patients. This bias, if present, could influence the observed risk associated with HIV infection. However, it is unlikely that such a bias could account for the entire difference between groups, given the magnitude of the elevation in incidence rates in the HIV-infected population.

### Acknowledgments

The authors thank Sue Shallow, Gretchen Rothrock, Pam Daily, Nandeeni Murkerjee, and Lisa Gelling for their disease surveillance work.

This work was funded in part by Centers for Disease Control and Prevention cooperative agreement number U50/ CCU915546-01.

Mr. Baer is a 3rd-year medical student at the University of North Carolina at Chapel Hill. His current research includes examining a possible molecular pathway for the *Chlamydia pneumoniae* and atherosclerosis association.

### References

1. Bennett JV, Holmberg SD, Rogers MF, Solomon SL. Infectious and parasitic diseases. Am J Prev Med 1987;3:102-14.
2. Weissman JB, Schmerler A, Weiler P, Filice G, Godbey N, Hansen I. The role of preschool children and day-care centers in the spread of shigellosis in urban communities. J Pediatr 1974;84:797-802.
3. Ryan MJ, Wall PG, Adak GK, Evans HS, Cowden JM. Outbreaks of infectious intestinal disease in residential institutions in England and Wales 1992-1994. J Infect 1997;34:49-54.
4. DuPont HL, Gangarosa EJ, Reller LB, Woodward WE, Armstrong RW, Hammond J,et al. Shigellosis in custodial institutions. Am J Epidemiol 1970;92:172-9.
5. Dritz SK, Back AF. Shigella enteritis venereally transmitted [letter]. N Engl J Med 1974;291:1194.
6. Bader M, Petersen AH, Williams R, Spearman J, Anderson H. Venereal transmission of shigellosis in Seattle-King County. Sex Transm Dis 1977;4:89-91.
7. Dritz SK, Ainsworth TE, Garrard WF, Back A, Palmer RD, Boucher LA, et al. Patterns of sexually transmitted enteric diseases in a city. Lancet 1977;2:3-4.
8. Tauxe RV, McDonald RC, Hargrett-Bean N, Blake P. The persistence of *Shigella flexneri* in the United States: Increasing role in adult males. Am J Public Health 1988;78:1432-5.
9. Quinn TC, Stamm WE, Goodell SE, Mkrtichian E, Benedetti J, Corey L, et al. The polymicrobial origin of intestinal infections in homosexual men. N Engl J Med 1983;309:576-82.
10. Angulo FJ, Swerdlow DL. Bacterial enteric infections in persons infected with human immunodeficiency virus. Clin Infect Dis 1995;21 Suppl 1:S84-93.
11. Sorvillo FJ, Lieb LE, Waterman SH. Incidence of campylobacteriosis among patients with AIDS in Los Angeles County. J Acquir Immune Defic Syndr Hum Retrovirol 1991;4:598-602.
12. Celum CL, Chaisson RE, Rutherford GW, Barnhart JL, Echenberg DF. Incidence of salmonellosis in patients with AIDS. J Infect Dis 1987;156:998-1002.
13. U.S. Census Bureau. Annual time series of county population estimates by age, sex, race, and Hispanic origin; 1996; [accessed 1998 Aug 1]. Available from: URL http://www.census.gov/population/estimates/countypop.html.
14. Shafer KP, McFarland W, Katz MH. 1997 consensus report on HIV prevalence and incidence in San Francisco. San Francisco Department of Public Health HIV Seroepidemiology Unit 1997.
15. Centers for Disease Control and Prevention. Revised FoodNet 1996 final report. 1998 Sep.
16. Gaudio PA, Sethabutr O, Echeverria P, Hoge CW. Utility of a polymerase chain reaction diagnostic system in a study of the epidemiology of shigellosis among dysentery patients, family contacts, and well controls living in a shigellosis-endemic area. J Infect Dis 1997;176:1013-8.

